# Crystal structure of μ_3_-tetra­thio­anti­monato-tris[(cyclam)zinc(II)] tetra­thio­anti­monate aceto­nitrile disolvate dihydrate showing Zn disorder over the cyclam ring planes (cyclam = 1,4,8,11-tetra­aza­cyclo­tetra­deca­ne)

**DOI:** 10.1107/S2056989022003759

**Published:** 2022-04-12

**Authors:** Christian Näther, Felix Danker, Wolfgang Bensch

**Affiliations:** a Institut für Anorganische Chemie, Universität Kiel, Max-Eyth. Str. 2, 241128 Kiel, Germany

**Keywords:** crystal structure, Zn-thio­anti­monate, cation disorder, hydrogen bonding

## Abstract

Reaction of Zn(ClO_4_)_2_·6H_2_O with cyclam (cyclam = 1,4,8,11-tetra­aza­cyclo­tetra­decane, C_10_H_24_N_4_) and Na_3_SbS_4_ in an aceto­nitrile/water mixture led to the formation of crystals of the title compound, [Zn_3_(SbS_4_)(C_10_H_24_N_4_)_3_](SbS_4_)·2CH_3_CN·2H_2_O or [(Zn-cyclam)_3_(SbS_4_)_2_](H_2_O)_2_(aceto­nitrile)_2_. The set-up of the crystal structure is similar to that of [(Zn-cyclam)_3_(SbS_4_)_2_]^.^8H_2_O reported recently [Danker *et al.* (2021 ▸). *Dalton Trans*. **50**, 18107–18117]. The crystal structure of the title compound consists of three crystallographically independent Zn^II^ cations (each disordered around centers of inversion), three centrosymmetric cyclam ligands, one SbS_4_^3–^ anion, one water and one aceto­nitrile mol­ecule occupying general positions. The aceto­nitrile mol­ecule is equally disordered over two sets of sites. Each Zn^2+^ cation is bound to four nitro­gen atoms of a cyclam ligand and one sulfur atom of the SbS_4_^3–^ anion within a distorted square-pyramidal coordination. The cation disorder of the [Zn(cyclam)]^2+^ complexes is discussed in detail and is also observed in other compounds, where identical ligands are located above and below the [Zn(cyclam)]^2+^ plane. In the title compound, the building units are arranged in layers parallel to the *bc* plane forming pores in which the aceto­nitrile solvate mol­ecules are located. Inter­molecular C—H⋯S hydrogen bonding links these units to the SbS_4_^3–^ anions. Between the layers, additional water solvate mol­ecules are present that act as acceptor and donor groups for inter­molecular N—H⋯O and O—H⋯S hydrogen bonding.

## Abstract 2

In the crystal structure of the title compound, [Zn(cyclam)]^2+^ cations and SbS_4_^3–^ anions are present, which are linked to aceto­nitrile and water solvate mol­ecules *via* inter­molecular hydrogen bonding.

## 1. Chemical context

For several years, chalcogenidometallates and chalcogenides with inorganic and/or organic cations have been investigated intensively because several of them show promising physical properties (Feng *et al.*, 2021 ▸; Lokhande *et al.*, 2019 ▸; Thiele *et al.*, 2017 ▸; Feng *et al.*, 2016 ▸; Si *et al.*, 2016 ▸; Bensch & Kanatzidis, 2012 ▸). Hence, numerous such compounds have been reported in the literature (Sheldrick & Wachhold, 1998 ▸; Bensch *et al.*, 1997 ▸; Dehnen & Melullis, 2007 ▸; Wang *et al.*, 2016 ▸; Zhou, 2016 ▸; Zhu & Dai, 2017 ▸; Nie *et al.*, 2017 ▸). An important class of chalcogenidometallates are represented by thio­anti­monates, which exhibit a pronounced structural variability with different coordination numbers of the Sb^V^ atom and networks of different dimensionality (Spetzler *et al.*, 2004 ▸; Jia *et al.*, 2004 ▸; Powell *et al.*, 2005 ▸; Engelke *et al.*, 2004 ▸; Zhang *et al.*, 2007 ▸; Liu & Zhou, 2011 ▸), with some of them having potential for future applications (Zhou *et al.*, 2019 ▸).

For several years, we have been inter­ested in the syntheses and structural behaviors of thio­anti­monate(V) compounds (Stähler *et al.*, 2001 ▸; Schur *et al.*, 2001 ▸; Pienack *et al.*, 2008 ▸). In the early stages of these studies, such compounds were prepared at elevated temperatures under solvothermal conditions but subsequently, new synthetic approaches using soluble precursors such as Na_3_SbS_4_·9H_2_O were developed, which allowed the synthesis of new thio­anti­monates at room temperature (Anderer *et al.*, 2016 ▸). The major advantage of this route is that, under these conditions, thio­anti­monate compounds containing Sb^V^ atoms can be prepared selectively, which is otherwise difficult to achieve. In most cases, we used transition-metal complexes (TMCs) as counter-cations. In this context, cyclam (cyclam = 1,4,8,11-tetra­aza­cyclo­tetra­deca­ne) became of inter­est as a ligand. The formed complex cations are in a fourfold coordination environment and provide additional coordination sites for thio­anti­monate anions, which can lead to the formation of networks by (TMC)—S bonds to the anion. Following this synthetic approach, we reacted cyclam with Na_3_SbS_4_·9H_2_O and different transition-metal salts, which led to the formation of compounds with compositions: [(Cu-cyclam)_3_(SbS_4_)_2_]·20H_2_O, [(Zn-cyclam)_3_(SbS_4_)_2_]·8H_2_O (Danker *et al.*, 2021 ▸) and [(Co-cyclam)_3_(SbS_4_)_2_](H_2_O)_2_(aceto­nitrile)_2_ (Näther *et al.*, 2022 ▸). In the crystal structure of the cobalt and copper compounds, the metal cations are octa­hedrally coordinated by the four N atoms of the cyclam ligand and by two S atoms of the tetra­thio­anti­monate(V) anions in *trans*-positions. Each of the SbS_4_^3–^ anions coordinates to three crystallographically independent [*M*(cyclam)]^2+^ cations (*M* = Cu and Co), linking the cations and anions into layers. Within these layers, channels are formed in which water or aceto­nitrile solvate mol­ecules are located. These layers are connected into a three-dimensional network by inter­molecular hydrogen bonding *via* water mol­ecules. In the case of *M* = Zn, a different coordination is observed, because this cation is shifted out of the N_4_ plane of the cyclam ligand and because of the center of inversion is disordered over both ring planes (Danker *et al.*, 2021 ▸). In this context, it is noted that such a disorder in Zn–cyclam complexes has already been observed in other, different compounds, but the structural consequences were not discussed in detail (see *Database survey*). 
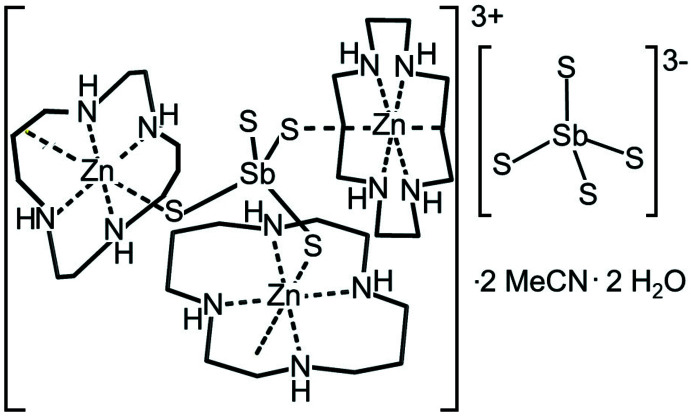


To find more examples of similar compounds, additional syntheses were performed. From an aceto­nitrile/water mixture, crystals of another Zn^II^–cyclam tetra­thio­anti­monate(V) compound with composition [(Zn-cyclam)_3_(SbS_4_)_2_](H_2_O)_2_(aceto­nitrile)_2_ were obtained. Likewise, in this compound disorder of the Zn^2+^ cations is observed and the structural consequences are discussed in this contribution.

## 2. Structural commentary

**Figure 1:**
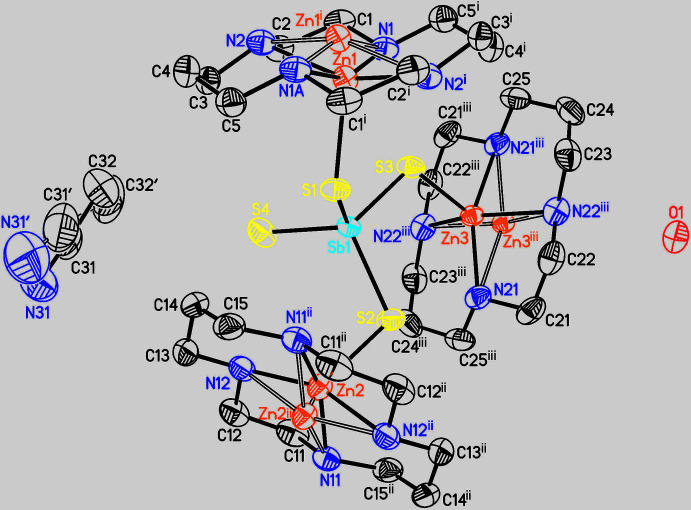
Part of the crystal structure of the title compound with labeling and displacement ellipsoids drawn at the 50% probability level. The hydrogen atoms were omitted for clarity; the disorder of the aceto­nitrile solvent mol­ecule and the Zn^2+^ cations is shown with full and open bonds. [Symmetry codes: (i) −*x* + 1, −*y*, −*z* + 1; (ii) −*x* + 1, −*y* + 1, −*z*; (iii) −*x*, −*y* + 1, −*z* + 1.]

**Figure 2:**
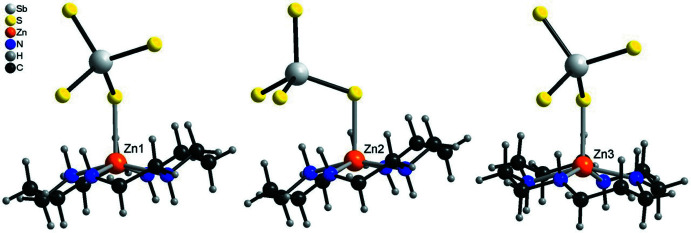
View of the coordination spheres of the three crystallographically independent Zn^2+^ cations. The cation disorder is not shown for clarity

**Figure 3:**
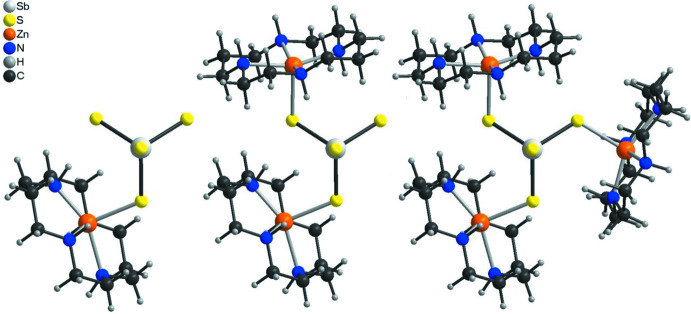
View of the three possible coordination modes of the SbS_4_^3–^ anion. The symmetry-equivalent Zn^2+^ cations generated by the center of inversion are not shown for clarity.

The asymmetric unit of the title compound consists of three half cyclam ligands (completed by inversion symmetry), one SbS_4_^3–^ anion, one water solvent mol­ecule, one disordered aceto­nitrile solvent mol­ecule and three Zn^2+^ cations that are disordered around centers of inversion (Fig. 1 ▸). In contrast to [(Cu-cyclam)_3_(SbS_4_)_2_]·20H_2_O (Danker *et al.*, 2021 ▸) and [(Co-cyclam)_3_(SbS_4_)_2_](H_2_O)_2_(aceto­nitrile)_2_ (Näther *et al.*, 2022 ▸), in both of which the cations are located at the center of the cyclam ligand and have an octa­hedral coordination, in the title compound the Zn^2+^ cations are shifted out of the N_4_ plane of the ligand by 0.4318 (6) Å (Zn1), 0.3751 (6) Å (Zn2) and 0.4998 (7) Å (Zn3). This means that each Zn^2+^ cation is in a fivefold coordination defined by the four N atoms of the cyclam ligand in the basal plane and one S atom of the SbS_4_^3–^ anions in the apical position (Fig. 2 ▸, Table 1 ▸). The Zn—S distances to the Zn^2+^ cation on the other side of the N_4_ plane are 3.2748 (8) Å (Zn1), 3.2063 (9) Å (Zn2) and 3.4234 (9) Å (Zn3), which are much too long for a significant inter­action. Because all of the Zn^2+^ cations are disordered around centers of inversion, the connectivity within the crystal structure is difficult to define. In principle, the SbS_4_^3–^ anions can coordin­ate to one, two or three [Zn(cyclam]^2+^ cations (Fig. 3 ▸).

**Figure 4:**
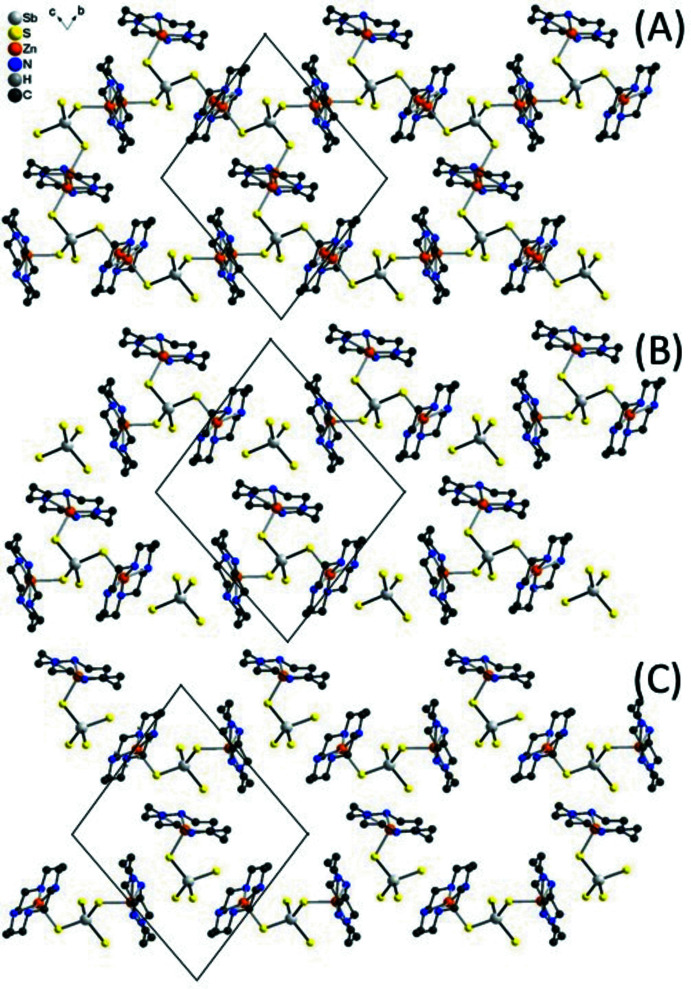
Crystal structure of the title compound showing the [(Zn-cyclam)_3_(SbS_4_)_2_] substructure with disorder of the Zn^2+^ cations (A), and assuming that an equivalent amount of [Zn(cyclam)_3_(SbS_4_)]^3+^ and [SbS_4_]^3−^ (B) or [Zn(cyclam)SbS_4_]^−^ and [(Zn(cyclam))_2_(SbS_4_)]^+^ units are present (C).

If the disorder were not present and the Zn^2+^ cations were located on centers of inversion in the planes of the cyclam ligands, layers would be formed (Fig. 4 ▸A) like in [(Cu-cyclam)_3_(SbS_4_)_2_]·20H_2_O (Danker *et al.*, 2021 ▸) or [(Co-cyclam)_3_(SbS_4_)_2_](H_2_O)_2_(aceto­nitrile)_2_ (Näther *et al.*, 2022 ▸) reported recently. In the case of [(Zn-cyclam)_3_(SbS_4_)_2_](H_2_O)_2_(aceto­nitrile)_2_, one can argue that each of the SbS_4_^3–^ anions acts as a tri-coordinating ligand like in the Cu and Co compounds and is connected to each of the [Zn(cyclam]^2+^ cations, forming [(Zn-cyclam)_3_(SbS_4_)]^3+^ moieties. However, in this case, an equivalent amount of non-coordinating SbS_4_^3–^ anions must be present for charge balance as well as for the correct ratio between Zn-cyclam cations and tetra­thio­anti­monate anions (Fig. 4 ▸B). Alternatively, the anion can coordinate to two cations forming [(Zn-cyclam)_2_(SbS_4_)]^+^ cations. Then, an equivalent amount of [(Zn-cyclam)(SbS_4_)]^−^ anions must be present to have the correct ratio between Zn-cyclam and the tetra­thio­anti­monate anions (Fig. 4 ▸C). The arrangement with [(Zn-cyclam)_3_(SbS_4_)]^3+^ cations and an SbS_4_^3–^ anion appears to be more likely because of the higher charge, but this is in fact difficult to prove. This possibility can also not be verified from the Sb—S bond lengths because they are very similar for the thio­anti­monate anions, which is expected because they are averaged over the whole crystal structure (Table 1 ▸).

It is noted that such a cation disorder is also observed in other compounds containing [Zn(cyclam)]^2+^ cations, which includes [(Zn-cyclam)_3_(SbS_4_)_2_]·8H_2_O (Danker *et al.*, 2021 ▸) and other compounds where identical anions are located above and below the N_4_ plane of the [Zn(cyclam]^2+^ cations (see *Database survey*). The reason for this disorder is still unclear. For [(Cu-cyclam)_3_(SbS_4_)_2_]·20H_2_O and [(Zn-cyclam)_3_(SbS_4_)_2_]·8H_2_O, DFT calculations were performed, which reasonably reproduced the octa­hedral coordination for the Cu^2+^ and the square-pyramidal coordination for the Zn^2+^ cations (Danker *et al.*, 2021 ▸). Moreover, these calculations also revealed that in the Cu compound, the attractive dispersion inter­actions between the cyclam ligand and the SbS_4_^3–^ anion contribute to the environment of the metal cation, which might be the reason for the different behavior of the Cu^2+^ and the Zn^2+^ cations. Also, for very large cations it might be possible that they are shifted out of the center of the cyclam ring, because there is not enough space available within the ring plane. To examine whether the size of Zn^2+^ might be a reason for the shift out of the N_4_ plane, we analyzed the ionic radii (Shannon, 1976 ▸) and found no significant differences for octa­hedrally coordinated Zn^2+^ (*r* = 0.74 Å), Co^2+^_hs_ (*r* = 0.745 Å), Co^2+^_ls_ (*r* = 0.65 Å) and Cu^2+^ (*r* = 0.73 Å). One may argue that in [(Co-cyclam)_3_(SbS_4_)_2_](H_2_O)_2_(aceto­nitrile)_2_, for which the spin state is not known, Co^2+^ is ordered because it adopts the low-spin state with a smaller ionic radius compared to the high-spin state. However, in [(Cu-cyclam)_3_(SbS_4_)_2_]·20H_2_O, no disorder is observed and the ionic radius of Cu^2+^ is similar to that of Zn^2+^, and larger than for Co^II^_ls_. Hence, the ionic radius is most probably not the driving force of the disorder of Zn^2+^. We also checked many other transition-metal cations in the form of their cyclam complexes, and there were no indications for metal disorder except in some of the Zn compounds, which suggests that such a disorder is limited to Zn^2+^ cations. Even for these compounds, only about 10% show disorder (see *Database survey*). A possible explanation for these observations might be the ligand field stabilization energy, which is zero for Zn^2+^ (electronic configuration 3*d* ^10^), while it is reasonably large for Co^2+^ (3*d* ^7^) and Cu^2+^ (3*d* ^9^), resulting in a preference of the position of these cations within the N_4_ plane. Because not all [Zn(cyclam]^2+^ complexes show disorder, secondary effects (sterical demands, packing) may also be responsible for the disorder.

## 3. Supra­molecular features

**Figure 5:**
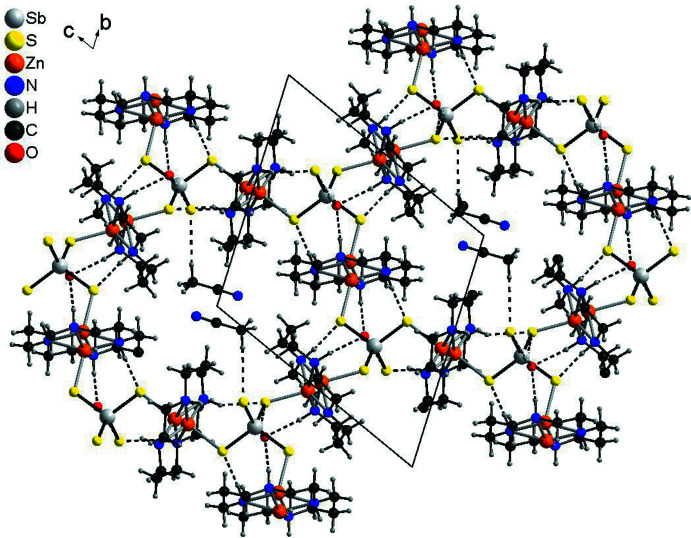
Crystal structure of the title compound in a view along the *a* axis with inter­molecular hydrogen bonding shown as dashed lines. The disorder of the aceto­nitrile mol­ecules is omitted for clarity, whereas that of each Zn^2+^ cation is indicated.

The cations and anions are arranged into layers parallel to the *bc* plane in such a way that channels are formed in which the disordered aceto­nitrile solvate mol­ecules are located. The latter are hydrogen-bonded to the tetra­thio­anti­monate anions by inter­molecular C—H⋯S inter­actions (Figs. 4 ▸ and 5 ▸, Table 2 ▸). One of the C—H⋯S angles is close to linearity, which indicates that this is a relatively strong inter­action. The water mol­ecules are located between the layers and are hydrogen-bonded to the tetra­thio­anti­monate anions *via* comparatively strong inter­molecular O—H⋯S inter­actions (Table 2 ▸). The water mol­ecules also act as acceptors for strong N—H⋯O hydrogen bonding involving the NH hydrogen atoms of the cyclam ligands (Fig. 5 ▸, Table 2 ▸). The NH groups are also hydrogen-bonded to the S atoms of the tetra­thio­anti­monate(V) groups. There are additional C—H⋯S inter­actions, but according to the the corresponding angles, it seems that these are only weak (Table 2 ▸).

## 4. Database survey

A search for structures of Zn^2+^–cyclam complexes in the Cambridge Structural Database (CSD version 5.42, last update November 2020; Groom *et al.*, 2016 ▸) led to 34 hits but none of them contains SbS_4_^3–^ anions. However, as mentioned above, one compound with composition [(Zn-cyclam)_3_(SbS_4_)_2_]·8H_2_O has already been reported (Danker *et al.*, 2021 ▸) but so far is not included in the database.

In one of the other structures, two [Zn(cyclam]^2+^ cations are linked by oxalate anions into a centrosymmetric dimer, which means that both O atoms are on the same side of one cyclam ring (FIHYEB; Jo *et al.*, 2005 ▸). In all remaining structures, the Zn^2+^ cations seem to be sixfold coordinated with one monocoordinating donor atom at each side of the cyclam ring; however, for five of them no atomic coordinates are given (HEGNEM10, HEGNOW, HEGNOW10, VUSDUI20 and WARJAD). For these hits, it is difficult to decide whether disorder is present or not. In some of the entries, the Zn disorder is mentioned in the database and this includes structures with the following refcodes: CUZHUA (Kato & Ito, 1985 ▸), in which the Zn^2+^ cations are coordinated by methyl­carbonato anions from both sites, DITZIP (Ito *et al.*, 1984 ▸), in which the cations are linked to two thio­cyanate anions and HEGNEM, HEGNOW and VUSDUI10 (Porai-Koshits *et al.*, 1994 ▸), in which chloride, bromide and iodine anions are located on each side of the cyclam ligand. It should be noted that, for the first structure determination of ZnCl_2_(cyclam) (VUSDUI; Antsyshkina *et al.*, 1991 ▸), no disorder is mentioned. One can assume that the disorder was overlooked and the Zn^2+^ cation forced to be situated at the center of inversion. For the remaining structures, the two Zn—*X* bond lengths (*X* = O, Cl, Br, I) are identical in each case, which points to ordered structures. Nonetheless, in some cases the Zn^2+^ cations are located on special positions and because no anisotropic displacement parameters are available in the corresponding CIFs, one cannot decide whether there are hints of disorder.

## 5. Synthesis and crystallization

**Synthesis of Na_3_SbS_4_·9H_2_O (Schlippe’s salt)**

Na_3_SbS_4_·9H_2_O was synthesized by adding 16.6 g (0.213 mol) of Na_2_S·*x*H_2_O (technical grade, purchased from Acros Organics) to 58 ml of demineralized water. This solution was heated to 333 K for 1h and afterwards 19.6 g (0.058 mol) of Sb_2_S_3_ (98%, purchased from Alfa Aesar) and 3.69 g (0.115 mol) of sulfur (min. 99%, purchased from Alfa Aesar) were added. The reaction mixture was then heated to 343 K for 6 h, filtered off and the filtrate was stored overnight at room temperature. Light-yellow-colored crystals formed overnight, were filtered off, washed with small amounts of water and dried *in vacuo*.

**Synthesis of tris­(cyclam-zinc(II)-bis-tetra­thio­anti­monate)-bis water-bis-aceto­nitrile solvate**

Single crystals of the title compound were serendipitously obtained by dissolving 10 mg (0.274 mmol) of Zn(ClO_4_)_2_·6H_2_O (purchased from Alfa Aesar) and 10 mg (0.05 mmol) of cyclam (purchased from Strem Chemicals) in 2 ml of aceto­nitrile (purchased from Merck) to which 20 mg (0.14 mmol) of Na_3_SbS_4_·9H_2_O dissolved in 1 ml of water were added. After storing this mixture for 3d at room temperature, a few colorless crystals of the title compound were obtained.

## 6. Refinement

Crystal data, data collection and structure refinement details are summarized in Table 3 ▸. The C—H and N—H hydrogen atoms were positioned with idealized geometry (methyl H atoms allowed to rotate but not to tip) and were refined with *U*_iso_(H) =1.2*U*_eq_(C,N) (1.5 for methyl H atoms) using a riding model. The water hydrogen atoms were located in a difference-Fourier map, and their bond lengths set to ideal values with *U*_iso_(H) = 1.5*U*_eq_(O) using a riding model. The acetontrile mol­ecule was modeled as being equally disordered over two sets of sites and was refined using a split model with restraints for the geometry and the components of the anisotropic displacement parameters. Each of the three Zn^2+^ cations was found to be disordered around a center of inversion and thus was refined with half occupancy.

The crystal structure was alternatively refined in space group *P*1 but the disorder remained the same. There were also no hints of superstructure reflections, and in the diffraction pattern diffuse scattering was not observed.

## Supplementary Material

Crystal structure: contains datablock(s) I. DOI: 10.1107/S2056989022003759/wm5639sup1.cif

Structure factors: contains datablock(s) I. DOI: 10.1107/S2056989022003759/wm5639Isup2.hkl

CCDC reference: 2164599

Additional supporting information:  crystallographic information; 3D view; checkCIF report

## Figures and Tables

**Table 1:** Selected bond lengths (Å)

Sb1—S4	2.3049 (7)	Zn1—N2^i^	2.039 (2)
Sb1—S2	2.3214 (7)	Zn1—N2	2.196 (2)
Sb1—S3	2.3252 (6)	Zn2—N12	2.048 (2)
Sb1—S1	2.3358 (6)	Zn2—N11^ii^	2.052 (2)
S1—Zn1	2.4071 (8)	Zn2—N11	2.179 (2)
S2—Zn2	2.4614 (9)	Zn3—N21	2.020 (2)
S3—Zn3	2.4300 (8)	Zn3—N22^iii^	2.043 (2)
Zn1—N1	2.028 (2)	Zn3—N22	2.205 (2)

Symmetry codes: (i) 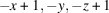 ; (ii) 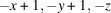 ; (iii) 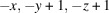 .

**Table 2:** Hydrogen-bond geometry (Å, °)

*D*—H⋯*A*	*D*—H	H⋯*A*	*D*⋯*A*	*D*—H⋯*A*
N1—H1⋯S3	1.00	2.39	3.380 (2)	172
N2—H2⋯S1^i^	1.00	2.78	3.400 (2)	121
N2—H2⋯O1^iv^	1.00	2.26	3.133 (3)	146
C3—H3*A*⋯S1	0.99	2.93	3.626 (3)	128
C5—H5*B*⋯S1	0.99	2.95	3.590 (3)	123
N11—H11⋯S1^ii^	1.00	2.49	3.433 (2)	157
N11—H11⋯S2^ii^	1.00	2.97	3.545 (2)	117
C11—H11*A*⋯S4^v^	0.99	2.96	3.872 (3)	154
N12—H12⋯S4	1.00	2.50	3.475 (2)	166
C13—H13*A*⋯S2^ii^	0.99	2.81	3.490 (3)	126
C15—H15*B*⋯S2^ii^	0.99	2.82	3.495 (3)	126
N21—H21⋯S2	1.00	2.29	3.287 (2)	172
N22—H22⋯S3^iii^	1.00	2.86	3.518 (2)	124
N22—H22⋯O1	1.00	2.18	2.940 (3)	131
C23—H23*B*⋯S3	0.99	3.01	3.670 (3)	125
C25—H25*A*⋯S3	0.99	2.86	3.547 (3)	127
O1—H1*O*⋯S1^vi^	0.84	2.52	3.286 (2)	152
O1—H2*O*⋯S4^iii^	0.84	2.47	3.305 (2)	173
C32—H32*A*⋯S4	0.98	2.96	3.92 (3)	170
C32′—H32*D*⋯S4	0.98	2.89	3.66 (3)	136

Symmetry codes: (i) 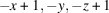 ; (ii) 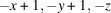 ; (iii) 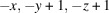 ; (iv) 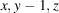 ; (v) 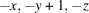 ; (vi) 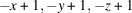 .

**Table 3:** Experimental details

Crystal data	
Chemical formula	[Zn_3_(SbS_4_)(C_10_H_24_N_4_)_3_](SbS_4_)·2CH_3_CN·2H_2_O
*M*_r_	1415.22
Crystal system, space group	Triclinic, *P* 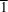
Temperature (K)	200
*a*, *b*, *c* (Å)	8.7856 (3), 13.1738 (6), 14.0096 (6)
α, β, γ (°)	67.018 (3), 77.677 (3), 84.220 (3)
*V* (Å^3^)	1458.10 (11)
*Z*	1
Radiation type	Mo *K*α
μ (mm^−1^)	2.46
Crystal size (mm)	0.16 × 0.12 × 0.09

Data collection	
Diffractometer	Stoe IPDS2
Absorption correction	Numerical (*X-RED* and *X-SHAPE*; Stoe, 2008 ▸)
*T*_min_, *T*_max_	0.562, 0.781
No. of measured, independent and observed [*I* > 2σ(*I*)] reflections	14357, 6303, 5594
*R*_int_	0.038
(sin θ/λ)_max_ (Å^−1^)	0.639

Refinement	
*R*[*F* ^2^ > 2σ(*F* ^2^)], *wR*(*F* ^2^), *S*	0.029, 0.077, 1.02
No. of reflections	6303
No. of parameters	327
No. of restraints	75
H-atom treatment	H-atom parameters constrained
Δρ_max_, Δρ_min_ (e Å^−3^)	0.81, −0.80

Computer programs: *X-AREA* (Stoe, 2008 ▸), *SHELXT* (Sheldrick, 2015*a* ▸), *SHELXL* (Sheldrick, 2015*b* ▸), *DIAMOND* (Brandenburg, 1999 ▸) and *publCIF* (Westrip, 2010 ▸).

## supplementary crystallographic information

### Crystal data

**Table:**

[Zn_3_(SbS_4_)(C_10_H_24_N_4_)_3_](SbS_4_)·2CH_3_CN·2H_2_O	*Z* = 1
*M**_r_* = 1415.22	*F*(000) = 720
Triclinic, *P*1	*D*_x_ = 1.612 Mg m^−^^3^
*a* = 8.7856 (3) Å	Mo *K*α radiation, λ = 0.71073 Å
*b* = 13.1738 (6) Å	Cell parameters from 14357 reflections
*c* = 14.0096 (6) Å	θ = 1.6–27.0°
α = 67.018 (3)°	µ = 2.46 mm^−^^1^
β = 77.677 (3)°	*T* = 200 K
γ = 84.220 (3)°	Block, colorless
*V* = 1458.10 (11) Å^3^	0.16 × 0.12 × 0.09 mm

### Data collection

**Table:**

Stoe IPDS-2 diffractometer	5594 reflections with *I* > 2σ(*I*)
ω scans	*R*_int_ = 0.038
Absorption correction: numerical (X-RED and X-SHAPE; Stoe, 2008)	θ_max_ = 27.0°, θ_min_ = 1.6°
*T*_min_ = 0.562, *T*_max_ = 0.781	*h* = −10→11
14357 measured reflections	*k* = −15→16
6303 independent reflections	*l* = −17→17

### Refinement

**Table:**

Refinement on *F*^2^	75 restraints
Least-squares matrix: full	Hydrogen site location: mixed
*R*[*F*^2^ > 2σ(*F*^2^)] = 0.029	H-atom parameters constrained
*wR*(*F*^2^) = 0.077	*w* = 1/[σ^2^(*F*_o_^2^) + (0.0533*P*)^2^] where *P* = (*F*_o_^2^ + 2*F*_c_^2^)/3
*S* = 1.02	(Δ/σ)_max_ < 0.001
6303 reflections	Δρ_max_ = 0.81 e Å^−^^3^
327 parameters	Δρ_min_ = −0.80 e Å^−^^3^

### Special details

**Table:**

Geometry. All esds (except the esd in the dihedral angle between two l.s. planes) are estimated using the full covariance matrix. The cell esds are taken into account individually in the estimation of esds in distances, angles and torsion angles; correlations between esds in cell parameters are only used when they are defined by crystal symmetry. An approximate (isotropic) treatment of cell esds is used for estimating esds involving l.s. planes.

### Fractional atomic coordinates and isotropic or equivalent isotropic displacement parameters (Å^2^)

**Table:**

	*x*	*y*	*z*	*U*_iso_*/*U*_eq_	Occ. (<1)
Sb1	0.25921 (2)	0.30825 (2)	0.30750 (2)	0.02516 (6)	
S1	0.48271 (7)	0.19527 (5)	0.31911 (5)	0.03152 (13)	
S2	0.35056 (8)	0.48478 (5)	0.20520 (5)	0.03581 (15)	
S3	0.13698 (8)	0.29796 (5)	0.47540 (5)	0.03425 (14)	
S4	0.09240 (8)	0.25656 (7)	0.22862 (6)	0.04413 (17)	
Zn1	0.49252 (6)	0.03467 (5)	0.47770 (4)	0.02883 (13)	0.5
N1	0.3114 (2)	0.05124 (19)	0.58800 (17)	0.0335 (5)	
H1	0.269523	0.126482	0.549863	0.040*	
C1	0.1818 (3)	−0.0192 (2)	0.6033 (2)	0.0351 (6)	
H1A	0.081605	0.016393	0.622325	0.042*	
H1B	0.191006	−0.090967	0.661824	0.042*	
C2	0.1848 (3)	−0.0376 (2)	0.5023 (2)	0.0357 (6)	
H2A	0.103121	−0.090218	0.514447	0.043*	
H2B	0.163021	0.032981	0.445923	0.043*	
N2	0.3388 (2)	−0.08173 (19)	0.46890 (18)	0.0336 (5)	
H2	0.356027	−0.158230	0.519687	0.040*	
C3	0.3665 (3)	−0.0776 (2)	0.3609 (2)	0.0368 (6)	
H3A	0.361440	0.000038	0.311335	0.044*	
H3B	0.283808	−0.118224	0.353400	0.044*	
C4	0.5251 (4)	−0.1280 (2)	0.3323 (2)	0.0425 (6)	
H4A	0.535867	−0.200472	0.389591	0.051*	
H4B	0.527222	−0.141658	0.267327	0.051*	
C5	0.6657 (3)	−0.0594 (2)	0.3143 (2)	0.0378 (6)	
H5A	0.760284	−0.092999	0.284689	0.045*	
H5B	0.651608	0.015681	0.261789	0.045*	
Zn2	0.47865 (8)	0.48973 (6)	0.02914 (4)	0.03109 (15)	0.5
N11	0.3674 (3)	0.64170 (19)	−0.06257 (18)	0.0355 (5)	
H11	0.420952	0.668281	−0.138314	0.043*	
C11	0.2113 (3)	0.6021 (3)	−0.0510 (2)	0.0435 (7)	
H11A	0.151576	0.660530	−0.098613	0.052*	
H11B	0.154414	0.583893	0.022484	0.052*	
C12	0.2264 (3)	0.5006 (3)	−0.0783 (2)	0.0424 (7)	
H12A	0.121747	0.471917	−0.068014	0.051*	
H12B	0.276978	0.519890	−0.153222	0.051*	
N12	0.3206 (3)	0.4154 (2)	−0.00964 (17)	0.0364 (5)	
H12	0.245116	0.382658	0.058312	0.044*	
C13	0.3676 (4)	0.3214 (3)	−0.0419 (2)	0.0435 (6)	
H13A	0.426065	0.348879	−0.115575	0.052*	
H13B	0.273100	0.285028	−0.039931	0.052*	
C14	0.4682 (4)	0.2372 (2)	0.0285 (2)	0.0457 (7)	
H14A	0.477149	0.169986	0.012069	0.055*	
H14B	0.413366	0.216727	0.102751	0.055*	
C15	0.6316 (4)	0.2725 (2)	0.0200 (2)	0.0426 (6)	
H15A	0.692015	0.207408	0.058677	0.051*	
H15B	0.683860	0.300684	−0.055150	0.051*	
Zn3	0.02379 (6)	0.47066 (5)	0.48481 (4)	0.02840 (12)	0.5
N21	0.1173 (3)	0.59830 (18)	0.35441 (18)	0.0367 (5)	
H21	0.179085	0.558725	0.310298	0.044*	
C21	0.2413 (3)	0.6496 (3)	0.3744 (3)	0.0447 (7)	
H21A	0.198038	0.712843	0.393720	0.054*	
H21B	0.322558	0.677280	0.309827	0.054*	
C22	0.3117 (3)	0.5647 (3)	0.4636 (3)	0.0451 (7)	
H22A	0.362021	0.504205	0.442243	0.054*	
H22B	0.392054	0.599273	0.480480	0.054*	
N22	0.1876 (3)	0.5206 (2)	0.5570 (2)	0.0389 (5)	
H22	0.140207	0.580173	0.582220	0.047*	
C23	0.2372 (4)	0.4264 (3)	0.6434 (3)	0.0471 (7)	
H23A	0.321898	0.449007	0.667048	0.056*	
H23B	0.278787	0.367155	0.617571	0.056*	
C24	0.1030 (4)	0.3821 (3)	0.7364 (2)	0.0523 (8)	
H24A	0.146591	0.329913	0.797575	0.063*	
H24B	0.054297	0.444461	0.755461	0.063*	
C25	−0.0237 (4)	0.3241 (2)	0.7196 (2)	0.0451 (7)	
H25A	0.024669	0.267581	0.691781	0.054*	
H25B	−0.092723	0.286099	0.788306	0.054*	
O1	0.2409 (3)	0.68506 (19)	0.63965 (19)	0.0483 (5)	
H1O	0.312801	0.692641	0.667254	0.073*	
H2O	0.159231	0.696791	0.678014	0.073*	
N31	0.179 (2)	0.0940 (13)	−0.0720 (10)	0.134 (6)	0.5
C31	0.164 (5)	0.059 (2)	0.0179 (13)	0.113 (7)	0.5
C32	0.131 (5)	0.013 (3)	0.1293 (14)	0.133 (11)	0.5
H32A	0.135202	0.070565	0.156711	0.199*	0.5
H32B	0.207310	−0.045371	0.155056	0.199*	0.5
H32C	0.026015	−0.017817	0.153554	0.199*	0.5
N31'	0.248 (3)	0.0336 (17)	−0.0501 (15)	0.171 (9)	0.5
C31'	0.174 (6)	0.030 (3)	0.0300 (19)	0.129 (9)	0.5
C32'	0.094 (4)	0.042 (3)	0.1232 (18)	0.130 (11)	0.5
H32D	0.156558	0.086324	0.142552	0.195*	0.5
H32E	0.077408	−0.030660	0.180249	0.195*	0.5
H32F	−0.006195	0.079325	0.111713	0.195*	0.5

### Atomic displacement parameters (Å^2^)

**Table:**

	*U*^11^	*U*^22^	*U*^33^	*U*^12^	*U*^13^	*U*^23^
Sb1	0.02327 (9)	0.02630 (9)	0.02246 (9)	0.00062 (6)	−0.00337 (6)	−0.00633 (6)
S1	0.0276 (3)	0.0294 (3)	0.0303 (3)	0.0036 (2)	−0.0045 (2)	−0.0051 (2)
S2	0.0441 (4)	0.0264 (3)	0.0292 (3)	−0.0021 (2)	0.0042 (3)	−0.0079 (2)
S3	0.0395 (3)	0.0293 (3)	0.0262 (3)	0.0066 (2)	0.0003 (2)	−0.0074 (2)
S4	0.0317 (3)	0.0659 (5)	0.0360 (3)	−0.0125 (3)	−0.0068 (3)	−0.0177 (3)
Zn1	0.0220 (2)	0.0361 (3)	0.0293 (3)	−0.0022 (3)	−0.0033 (2)	−0.0137 (2)
N1	0.0293 (10)	0.0340 (11)	0.0308 (11)	−0.0014 (8)	−0.0038 (8)	−0.0063 (9)
C1	0.0227 (11)	0.0336 (13)	0.0389 (14)	−0.0004 (9)	−0.0004 (10)	−0.0059 (11)
C2	0.0226 (11)	0.0369 (13)	0.0439 (14)	−0.0024 (10)	−0.0098 (10)	−0.0091 (11)
N2	0.0276 (10)	0.0355 (11)	0.0341 (11)	0.0024 (8)	−0.0095 (8)	−0.0083 (9)
C3	0.0419 (14)	0.0341 (13)	0.0390 (14)	−0.0048 (11)	−0.0140 (11)	−0.0146 (11)
C4	0.0517 (16)	0.0396 (15)	0.0423 (15)	−0.0002 (12)	−0.0094 (13)	−0.0219 (13)
C5	0.0395 (14)	0.0383 (14)	0.0327 (13)	0.0030 (11)	−0.0001 (11)	−0.0148 (11)
Zn2	0.0277 (4)	0.0299 (3)	0.0374 (4)	0.0002 (3)	−0.0105 (3)	−0.0125 (4)
N11	0.0339 (11)	0.0384 (12)	0.0301 (11)	0.0003 (9)	−0.0026 (9)	−0.0106 (9)
C11	0.0305 (13)	0.0547 (17)	0.0378 (14)	0.0074 (12)	−0.0101 (11)	−0.0097 (13)
C12	0.0294 (13)	0.0606 (18)	0.0354 (14)	−0.0069 (12)	−0.0106 (11)	−0.0127 (13)
N12	0.0360 (11)	0.0434 (13)	0.0283 (10)	−0.0075 (9)	−0.0054 (9)	−0.0108 (9)
C13	0.0527 (17)	0.0447 (16)	0.0352 (14)	−0.0171 (13)	−0.0045 (12)	−0.0155 (12)
C14	0.0658 (19)	0.0319 (13)	0.0358 (14)	−0.0117 (13)	−0.0005 (13)	−0.0110 (11)
C15	0.0533 (17)	0.0306 (13)	0.0364 (14)	0.0036 (12)	−0.0011 (12)	−0.0097 (11)
Zn3	0.0258 (3)	0.0273 (3)	0.0292 (3)	−0.0013 (2)	−0.0054 (2)	−0.0074 (2)
N21	0.0390 (12)	0.0287 (11)	0.0401 (12)	0.0007 (9)	−0.0036 (10)	−0.0128 (9)
C21	0.0372 (14)	0.0399 (15)	0.0529 (17)	−0.0132 (12)	0.0103 (12)	−0.0199 (13)
C22	0.0271 (13)	0.0532 (17)	0.0628 (19)	−0.0058 (12)	−0.0031 (12)	−0.0319 (15)
N22	0.0302 (11)	0.0382 (12)	0.0505 (14)	0.0054 (9)	−0.0093 (10)	−0.0198 (11)
C23	0.0439 (16)	0.0516 (17)	0.0567 (18)	0.0151 (13)	−0.0262 (14)	−0.0276 (15)
C24	0.071 (2)	0.0530 (18)	0.0367 (15)	0.0161 (16)	−0.0234 (15)	−0.0185 (14)
C25	0.0580 (18)	0.0358 (14)	0.0307 (13)	0.0060 (13)	−0.0015 (12)	−0.0062 (11)
O1	0.0433 (11)	0.0527 (13)	0.0576 (13)	−0.0048 (9)	−0.0073 (10)	−0.0303 (11)
N31	0.222 (18)	0.123 (11)	0.059 (5)	−0.092 (12)	0.002 (8)	−0.029 (7)
C31	0.197 (16)	0.087 (12)	0.067 (6)	−0.097 (12)	0.008 (9)	−0.036 (6)
C32	0.24 (3)	0.102 (16)	0.060 (6)	−0.043 (14)	−0.024 (11)	−0.030 (7)
N31'	0.25 (2)	0.146 (15)	0.112 (13)	−0.031 (14)	0.020 (12)	−0.070 (12)
C31'	0.183 (16)	0.110 (19)	0.112 (12)	−0.049 (15)	0.007 (11)	−0.067 (12)
C32'	0.18 (2)	0.119 (19)	0.109 (13)	−0.085 (17)	0.025 (13)	−0.071 (13)

### Geometric parameters (Å, º)

**Table:**

Sb1—S4	2.3049 (7)	N12—H12	1.0000
Sb1—S2	2.3214 (7)	C13—C14	1.518 (4)
Sb1—S3	2.3252 (6)	C13—H13A	0.9900
Sb1—S1	2.3358 (6)	C13—H13B	0.9900
S1—Zn1	2.4071 (8)	C14—C15	1.520 (5)
S2—Zn2	2.4614 (9)	C14—H14A	0.9900
S3—Zn3	2.4300 (8)	C14—H14B	0.9900
Zn1—N1	2.028 (2)	C15—H15A	0.9900
Zn1—N2^i^	2.039 (2)	C15—H15B	0.9900
Zn1—N2	2.196 (2)	Zn3—N21	2.020 (2)
Zn1—N1^i^	2.200 (2)	Zn3—N22^iii^	2.043 (2)
N1—C1	1.468 (3)	Zn3—N22	2.205 (2)
N1—C5^i^	1.471 (4)	Zn3—N21^iii^	2.207 (2)
N1—H1	1.0000	N21—C21	1.466 (4)
C1—C2	1.521 (4)	N21—C25^iii^	1.471 (4)
C1—H1A	0.9900	N21—H21	1.0000
C1—H1B	0.9900	C21—C22	1.515 (5)
C2—N2	1.474 (3)	C21—H21A	0.9900
C2—H2A	0.9900	C21—H21B	0.9900
C2—H2B	0.9900	C22—N22	1.471 (4)
N2—C3	1.461 (4)	C22—H22A	0.9900
N2—H2	1.0000	C22—H22B	0.9900
C3—C4	1.525 (4)	N22—C23	1.462 (4)
C3—H3A	0.9900	N22—H22	1.0000
C3—H3B	0.9900	C23—C24	1.522 (5)
C4—C5	1.524 (4)	C23—H23A	0.9900
C4—H4A	0.9900	C23—H23B	0.9900
C4—H4B	0.9900	C24—C25	1.517 (5)
C5—H5A	0.9900	C24—H24A	0.9900
C5—H5B	0.9900	C24—H24B	0.9900
Zn2—N12	2.048 (2)	C25—H25A	0.9900
Zn2—N11^ii^	2.052 (2)	C25—H25B	0.9900
Zn2—N12^ii^	2.172 (2)	O1—H1O	0.8400
Zn2—N11	2.179 (2)	O1—H2O	0.8400
N11—C11	1.470 (4)	N31—C31	1.142 (17)
N11—C15^ii^	1.471 (4)	C31—C32	1.41 (2)
N11—H11	1.0000	C32—H32A	0.9800
C11—C12	1.513 (5)	C32—H32B	0.9800
C11—H11A	0.9900	C32—H32C	0.9800
C11—H11B	0.9900	N31'—C31'	1.155 (19)
C12—N12	1.476 (3)	C31'—C32'	1.41 (2)
C12—H12A	0.9900	C32'—H32D	0.9800
C12—H12B	0.9900	C32'—H32E	0.9800
N12—C13	1.471 (4)	C32'—H32F	0.9800
			
S4—Sb1—S2	111.23 (3)	C13—N12—Zn2	120.73 (18)
S4—Sb1—S3	110.64 (3)	C12—N12—Zn2	109.48 (18)
S2—Sb1—S3	110.24 (2)	C13—N12—H12	103.4
S4—Sb1—S1	110.37 (3)	C12—N12—H12	103.4
S2—Sb1—S1	104.65 (2)	Zn2—N12—H12	103.4
S3—Sb1—S1	109.55 (2)	N12—C13—C14	112.6 (2)
Sb1—S1—Zn1	119.36 (3)	N12—C13—H13A	109.1
Sb1—S2—Zn2	108.53 (3)	C14—C13—H13A	109.1
Sb1—S3—Zn3	115.03 (3)	N12—C13—H13B	109.1
N1—Zn1—N2^i^	96.40 (9)	C14—C13—H13B	109.1
N1—Zn1—N2	83.55 (9)	H13A—C13—H13B	107.8
N2^i^—Zn1—N2	155.88 (4)	C13—C14—C15	116.4 (2)
N1—Zn1—N1^i^	155.90 (4)	C13—C14—H14A	108.2
N2^i^—Zn1—N1^i^	83.20 (9)	C15—C14—H14A	108.2
N2—Zn1—N1^i^	87.20 (9)	C13—C14—H14B	108.2
N1—Zn1—S1	106.20 (7)	C15—C14—H14B	108.2
N2^i^—Zn1—S1	99.42 (7)	H14A—C14—H14B	107.3
N2—Zn1—S1	103.76 (6)	N11^ii^—C15—C14	112.9 (2)
N1^i^—Zn1—S1	97.60 (6)	N11^ii^—C15—H15A	109.0
C1—N1—C5^i^	113.9 (2)	C14—C15—H15A	109.0
C1—N1—Zn1	111.15 (17)	N11^ii^—C15—H15B	109.0
C5^i^—N1—Zn1	122.20 (17)	C14—C15—H15B	109.0
C1—N1—H1	102.0	H15A—C15—H15B	107.8
C5^i^—N1—H1	102.0	N21—Zn3—N22^iii^	95.73 (10)
Zn1—N1—H1	102.0	N21—Zn3—N22	82.91 (10)
N1—C1—C2	109.7 (2)	N22^iii^—Zn3—N22	152.15 (4)
N1—C1—H1A	109.7	N21—Zn3—N21^iii^	152.13 (4)
C2—C1—H1A	109.7	N22^iii^—Zn3—N21^iii^	82.31 (10)
N1—C1—H1B	109.7	N22—Zn3—N21^iii^	86.13 (9)
C2—C1—H1B	109.7	N21—Zn3—S3	109.80 (7)
H1A—C1—H1B	108.2	N22^iii^—Zn3—S3	103.39 (7)
N2—C2—C1	109.8 (2)	N22—Zn3—S3	103.20 (6)
N2—C2—H2A	109.7	N21^iii^—Zn3—S3	97.61 (6)
C1—C2—H2A	109.7	C21—N21—C25^iii^	114.4 (2)
N2—C2—H2B	109.7	C21—N21—Zn3	111.54 (18)
C1—C2—H2B	109.7	C25^iii^—N21—Zn3	122.87 (19)
H2A—C2—H2B	108.2	C21—N21—H21	101.2
C3—N2—C2	113.9 (2)	C25^iii^—N21—H21	101.2
C3—N2—Zn1	109.35 (17)	Zn3—N21—H21	101.2
C2—N2—Zn1	100.71 (17)	N21—C21—C22	109.0 (2)
C3—N2—H2	110.8	N21—C21—H21A	109.9
C2—N2—H2	110.8	C22—C21—H21A	109.9
Zn1—N2—H2	110.8	N21—C21—H21B	109.9
N2—C3—C4	111.4 (2)	C22—C21—H21B	109.9
N2—C3—H3A	109.3	H21A—C21—H21B	108.3
C4—C3—H3A	109.3	N22—C22—C21	108.9 (2)
N2—C3—H3B	109.3	N22—C22—H22A	109.9
C4—C3—H3B	109.3	C21—C22—H22A	109.9
H3A—C3—H3B	108.0	N22—C22—H22B	109.9
C5—C4—C3	115.7 (2)	C21—C22—H22B	109.9
C5—C4—H4A	108.3	H22A—C22—H22B	108.3
C3—C4—H4A	108.3	C23—N22—C22	113.9 (2)
C5—C4—H4B	108.3	C23—N22—Zn3	111.25 (19)
C3—C4—H4B	108.3	C22—N22—Zn3	99.07 (18)
H4A—C4—H4B	107.4	C23—N22—H22	110.7
N1^i^—C5—C4	112.4 (2)	C22—N22—H22	110.7
N1^i^—C5—H5A	109.1	Zn3—N22—H22	110.7
C4—C5—H5A	109.1	N22—C23—C24	111.6 (2)
N1^i^—C5—H5B	109.1	N22—C23—H23A	109.3
C4—C5—H5B	109.1	C24—C23—H23A	109.3
H5A—C5—H5B	107.9	N22—C23—H23B	109.3
N12—Zn2—N11^ii^	95.95 (10)	C24—C23—H23B	109.3
N12—Zn2—N12^ii^	159.15 (4)	H23A—C23—H23B	108.0
N11^ii^—Zn2—N12^ii^	83.98 (10)	C25—C24—C23	116.2 (3)
N12—Zn2—N11	83.92 (9)	C25—C24—H24A	108.2
N11^ii^—Zn2—N11	159.22 (4)	C23—C24—H24A	108.2
N12^ii^—Zn2—N11	88.86 (9)	C25—C24—H24B	108.2
N12—Zn2—S2	102.88 (7)	C23—C24—H24B	108.2
N11^ii^—Zn2—S2	103.12 (7)	H24A—C24—H24B	107.4
N12^ii^—Zn2—S2	97.41 (7)	N21^iii^—C25—C24	111.9 (2)
N11—Zn2—S2	97.13 (7)	N21^iii^—C25—H25A	109.2
C11—N11—C15^ii^	114.2 (2)	C24—C25—H25A	109.2
C11—N11—Zn2	100.67 (17)	N21^iii^—C25—H25B	109.2
C15^ii^—N11—Zn2	111.02 (17)	C24—C25—H25B	109.2
C11—N11—H11	110.2	H25A—C25—H25B	107.9
C15^ii^—N11—H11	110.2	H1O—O1—H2O	103.9
Zn2—N11—H11	110.2	N31—C31—C32	174 (4)
N11—C11—C12	109.5 (2)	C31—C32—H32A	109.5
N11—C11—H11A	109.8	C31—C32—H32B	109.5
C12—C11—H11A	109.8	H32A—C32—H32B	109.5
N11—C11—H11B	109.8	C31—C32—H32C	109.5
C12—C11—H11B	109.8	H32A—C32—H32C	109.5
H11A—C11—H11B	108.2	H32B—C32—H32C	109.5
N12—C12—C11	109.4 (2)	N31'—C31'—C32'	171 (3)
N12—C12—H12A	109.8	C31'—C32'—H32D	109.5
C11—C12—H12A	109.8	C31'—C32'—H32E	109.5
N12—C12—H12B	109.8	H32D—C32'—H32E	109.5
C11—C12—H12B	109.8	C31'—C32'—H32F	109.5
H12A—C12—H12B	108.2	H32D—C32'—H32F	109.5
C13—N12—C12	114.1 (2)	H32E—C32'—H32F	109.5

Symmetry codes: (i) −*x*+1, −*y*, −*z*+1; (ii) −*x*+1, −*y*+1, −*z*; (iii) −*x*, −*y*+1, −*z*+1.

### Hydrogen-bond geometry (Å, º)

**Table:**

*D*—H···*A*	*D*—H	H···*A*	*D*···*A*	*D*—H···*A*
N1—H1···S3	1.00	2.39	3.380 (2)	172
N2—H2···S1^i^	1.00	2.78	3.400 (2)	121
N2—H2···O1^iv^	1.00	2.26	3.133 (3)	146
C3—H3*A*···S1	0.99	2.93	3.626 (3)	128
C5—H5*B*···S1	0.99	2.95	3.590 (3)	123
N11—H11···S1^ii^	1.00	2.49	3.433 (2)	157
N11—H11···S2^ii^	1.00	2.97	3.545 (2)	117
C11—H11*A*···S4^v^	0.99	2.96	3.872 (3)	154
N12—H12···S4	1.00	2.50	3.475 (2)	166
C13—H13*A*···S2^ii^	0.99	2.81	3.490 (3)	126
C15—H15*B*···S2^ii^	0.99	2.82	3.495 (3)	126
N21—H21···S2	1.00	2.29	3.287 (2)	172
N22—H22···S3^iii^	1.00	2.86	3.518 (2)	124
N22—H22···O1	1.00	2.18	2.940 (3)	131
C23—H23*B*···S3	0.99	3.01	3.670 (3)	125
C25—H25*A*···S3	0.99	2.86	3.547 (3)	127
O1—H1*O*···S1^vi^	0.84	2.52	3.286 (2)	152
O1—H2*O*···S4^iii^	0.84	2.47	3.305 (2)	173
C32—H32*A*···S4	0.98	2.96	3.92 (3)	170
C32′—H32*D*···S4	0.98	2.89	3.66 (3)	136

Symmetry codes: (i) −*x*+1, −*y*, −*z*+1; (ii) −*x*+1, −*y*+1, −*z*; (iii) −*x*, −*y*+1, −*z*+1; (iv) *x*, *y*−1, *z*; (v) −*x*, −*y*+1, −*z*; (vi) −*x*+1, −*y*+1, −*z*+1.
